# Effect of Recrystallization on β to α-Sn Allotropic Transition in 99.3Sn–0.7Cu wt. % Solder Alloy Inoculated with InSb

**DOI:** 10.3390/ma13040968

**Published:** 2020-02-21

**Authors:** Agata Skwarek, Balázs Illés, Tamás Hurtony, David Bušek, Karel Dušek

**Affiliations:** 1 Łukasiewicz Research Network—Institute of Electron Technology, Kraków Division, 30701 Krakow, Poland; askwarek@ite.waw.pl; 2 Department of Electrotechnology, Czech Technical University in Prague, 16627 Prague, Czech Republic; busekd1@fel.cvut.cz (D.B.); dusekk1@fel.cvut.cz (K.D.); 3 Department of Electronics Technology, Budapest University of Technology and Economics, 1111 Budapest, Hungary; hurtony@ett.bme.hu

**Keywords:** tin pest, recrystallization, grain size, β to α-Sn allotrope transition, SnAgCu (SAC), electrical resistance measurement

## Abstract

The effect of recrystallization of 99.3Sn–0.7Cu wt. % solder alloy on the allotropic transition of β to α-Sn (so-called tin pest phenomenon) was investigated. Bulk samples were prepared, and an InSb inoculator was mechanically applied to their surfaces to enhance the transition. Half of the samples were used as the reference material and the other half were annealed at 180 °C for 72 h, which caused the recrystallization of the alloy. The samples were stored at −10 and −20 °C. The β-Sn to α-Sn transition was monitored using electrical resistance measurements. The expansion and separation of the tin grains during the β-Sn to α-Sn transition process were studied using scanning electron microscopy. The recrystallization of the alloy suppressed the tin pest phenomenon considerably since it decreased the number of defects in the crystal structure where heterogeneous nucleation of β-Sn to α-Sn transition could occur. In the case of InSb inoculation, the spreading of the transition towards the bulk was as fast as the spreading parallel to the surface of the sample.

## 1. Introduction

Tin (Sn) is one of the most frequently used materials in the electronics industry. Sn is the base metal of solder alloys, surface finishes of solder pads, and component leads [1,2]. Furthermore, it is used as an optical layer [3] and as an anode in batteries [4]. Sn has two primary allotropes: α and β Sn. Engineering applications use the metallic β-Sn (white Sn), which has a body-centered tetragonal crystal structure in the space-group symmetry *I4*_1_/*amd*. α-Sn (gray Sn) is a semiconductor material with a diamond structure with cubic symmetry *Fd*$\bar{3}$*m*. There is also a considerable difference in mechanical properties between β and α-Sn (the α-Sn is fragile). The allotrope transition of β-Sn to α-Sn takes place in the case of cooling at 13.2 °C, and this phenomenon is the so-called “tin pest” [5]. The tin pest phenomenon is a degradation process of the tin and high tin content solder alloys. The transition results in a substantial volume increase (about 27%) [6] since the density of β-Sn is 7285 kg/m^3^, and the density of α-Sn is 5772 kg/m^3^ at 298 K [7].

The β-Sn to α-Sn transition process can be divided into the following characteristic stages: nucleation (transient), growth (after steady state nucleation), and in some cases, saturation (when the growth stops) [8]. During the nucleation stage, embryos form via self-organization, which later become nuclei (stable embryos) of the new phase. Homogeneous and heterogeneous types of nucleation can be distinguished. Homogeneous nucleation shows stochastic behavior without preferred nucleation sites. Heterogeneous nucleation occurs at defects (vacancies, stacking faults, dislocations) or impurities [9]. These sites act as catalysts (inoculants) of the nucleation of a phase transformation. Nucleation of a phase transformation can occur with a small driving force (e.g., undercooling or supersaturation). In the case of the β to α transition, the nucleation time is usually long. It can take most of the time of β to α transition (even years) [7]. 

**Figure 1:**
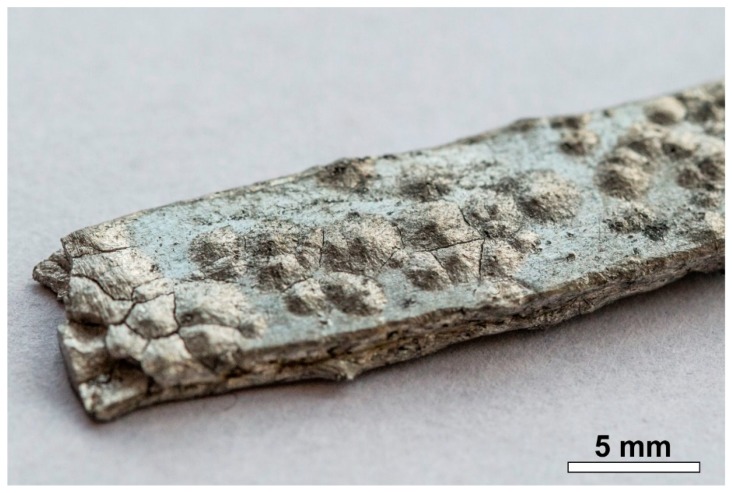
Tin pest warts and cracks on the surface of a tin solder bar; decomposition has already started at the ends of the bar.

In the growth stage, if the growth is not suppressed, nuclei start to grow, and a new stable or metastable phase is formed. Later, the metastable phase might or might not transform into a stable phase (according to the growth kinetics). The first visual signs of the transition appear as blemishes, discoloration, and mottling on the surface. Later, warts grow on the surface, from which the name “tin pest” originates. After the transition of a certain amount of a β-Sn, the specimen will decompose into powder; this is related to the volume increase and the low mechanical stability of the α-Sn (Figure 1).

The volume fraction of α-Sn formed from the phase transformation of β-Sn to α-Sn can vary significantly. In some cases, the transition stops after a while, which means that the volume fraction remains constant (saturation stage) [8], while in other cases, saturation is not detected [10]. The β to α-Sn transition is autocatalytic, which means that the growth kinetics depends on the volume fraction of α-Sn [11]. Generally, β to α-Sn transition can be described by Johnson–Mehl–Avrami kinetics with an Avrami exponent of 3 [7,11]. Furthermore, Oehl et al. [12] reported that in nanoscale, the growth kinetics of β to α-Sn transition also depends on the size of β-Sn particles.

At room conditions (20 °C/50 RH %), the tin pest is not believed likely to occur in the case of commercially available lead-free solder alloys [13]. However, identifying and describing tin pest is important for electronic appliances which need to work at sub-zero temperatures too, like aeronautical, aerospace and automobile applications [14]. The presence of materials with the same crystallographic structure and similar lattice parameters to α-Sn (like CdTe, InSb, and Ge) [9,15] or of low temperatures (under −30 °C) [16] can accelerate the kinetics of the transition. InSb and CdTe are widely used in microelectronics. The high electron mobility and small direct bandgap of InSb make it an attractive material for infrared optoelectronics and high-speed electronics [17], while CdTe is used in photovoltaic solar cells [18,19]. Therefore, these materials can come into contact with Sn-based solder joints. On the other hand, InSb and CdTe are intentionally used to enhance the transition as heterogeneous nucleation catalysts (a technique known as seeding or inoculation) when their powder is pressed into the β-Sn specimen [20]. 

The proximity of other metals can suppress the β-Sn to α-Sn transition. Zeng et al. [11,15] investigated the effect of adding 1 wt. % Pb, Cu, Ge, and Si powders and found that they all suppress the β→α transition. According to them, the primary mechanism of the additive was its close physical contact with Sn, which may hinder the advance of the β/α interface. However, there are some ambiguous results in previous studies regarding the effect of Cu because, as Skwarek et al. [14] showed, alloying 1 wt. % of Cu into Sn shortens the nucleation time of the transition. The alloying of electropositive metals (compared to Sn), which are soluble in the solid phase of Sn (like Sb, Pb, and Bi), suppresses the transition by decreasing the transition temperature [21]. Insoluble elements, such as Zn, Al, Mg, and Mn, have the opposite effect [22,23]. Regarding the most frequently used SnAgCu (SAC) solder alloys, Ag has the most significant impact on β→α transition. These studies agree that even a small amount of Ag (0.1 wt. %) suppresses the transition very effectively [24,25]. 

Wang et al. [4] achieved effective suppression of the tin pest phenomenon with different configurations of Sn and C films for the planar anode in Li-ion batteries. Using a C layer resulted in a considerably improved capacity retention rate and prevented the Sn film from cracking. Di Maio et al. [20] state that the residues of the fluxes used and the oxidation of the solder joints have a “natural suppression” effect on β-Sn to α-Sn transition. Zeng et al. [26] investigated the dependence of the kinetics of β-Sn to α-Sn transition on the annealing time at −45 °C. They found that an increase in the annealing time delayed the nucleation at −45 °C. The annealing of the solder joints at elevated temperature (>170 °C) has a positive effect on other types of failure mechanisms as well, like tin whisker growth. It has been claimed that the rearrangement and regrowth of the grains improve the stress relaxation ability of the solder joints and decrease grain boundary diffusion, which are the most essential factors in Sn whisker development [27]. As both phenomena are related to the Sn properties, it seems that recrystallization can have a significant influence on tin pest as well. Therefore, this study aims to investigate the effect of recrystallization on β-Sn to α-Sn transition in the case of 99.3Sn–0.7Cu wt. % solder alloys. 

## 2. Materials and Methods 

**Figure 2:**
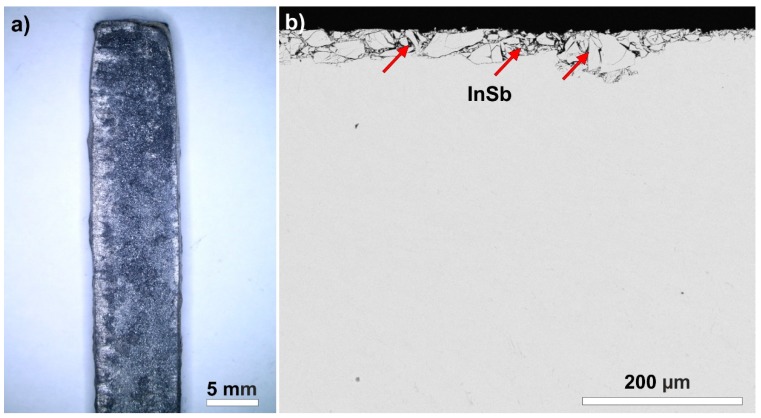
Optical micrographs of the sample: (**a**) the surface of a sample after the application of InSb; (**b**) penetration of InSb into the sample body.

The 99.3Sn–0.7Cu wt. % alloy (and its micro-alloyed variation) is widely used in the electronics industry [2]. Bulk samples were prepared by casting, according to the tin pest induction method developed by Skwarek et al. [28]. An amount of 10 g of the alloy was heated up to 300 °C and melted in a stainless-steel casting mold (45 × 6 × 3 mm^3^) under an air atmosphere. The samples were dipped in an HCl solution to remove oxides from the surface, which might be formed during the casting. InSb inoculator (with particle size: 1–10 µm) was applied to the surface of the samples with axial compression to enhance the transition (Figure 2a). The laminator was cleaned with isopropyl alcohol before the lamination process to avoid contamination of the samples. A mechanic laminator was used with a 30 kN force. The inoculator powder was pressed ~60–80 μm deep into the upper layer of the samples (Figure 2b), so it damaged the surface of the samples considerably, and resulted in strain energy stored in near-surface areas of the samples, which can enhance heterogeneous nucleation.

**Figure 3:**
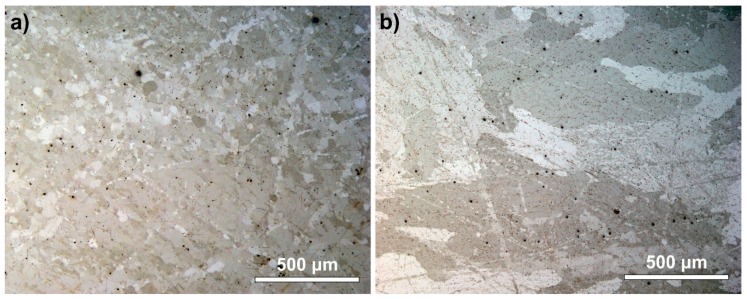
Optical micrographs of the microstructure of the samples: (**a**) reference; (**b**) annealed at 180 °C for 72 h.

The samples were divided into two groups. The first group was used as a reference. The second group was annealed at 180 °C for 72 h. The surface of the samples was examined by optical microscopy before and after the annealing, but no changes were found. The microstructure of the samples was also observed using polarized light microscopy after cross-sectioning. The surface of the cross-sections was treated with OPS (Oxide Polishing Suspension) to make the grain structure visible. Figure 3 shows the grain structure of the reference and annealed samples. The annealing resulted in recrystallization of the samples. The average grain size of the reference sample was ~50 μm, which was increased to ~500 μm during annealing. The black spots in the sample body are impurities, which are consequences of the casting process (and they usually occur in solder joints as well). The samples were stored at two different temperatures, −10 °C and −20 °C, for 40 and 20 weeks respectively. Fifteen samples were prepared from each sample type (totaling 60 samples altogether) for the tests.

The β-Sn to α-Sn transition was monitored using 4-probe electrical resistance measurements with an AGILENT 4338B milliohm meter (Santa Clara, CA, United States) at room temperature every 2 weeks. The measurement accuracy of the instrument at the mΩ range is under 3%. The repeatability error of the resistance measurements is under 2%. Initially, the average electrical resistance of the samples was 0.2 mΩ with 4% deviation, due to small differences in the sample sizes. Some of the samples were cross-sectioned before the tests. The aim was to study the expansion and separation of tin grains during the β-Sn to α-Sn transition and to observe the impact of recrystallization on the microstructure. The cross-sections were investigated using an Olympus BX-51 optical microscope (Olympus, Tokyo, Japan) and an FEI Inspect S50 Scanning Electron Microscope (SEM) (Hillsboro, Oregon, OR, USA).

## 3. Results and Discussions

**Figure 4:**
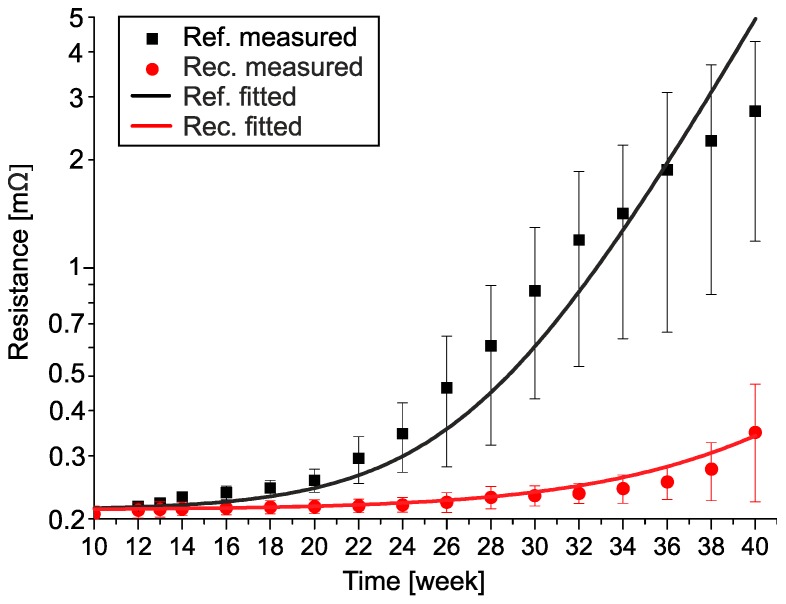
Plot of the electrical resistance changes in the reference (black) and recrystallized (red) samples at −10 °C, showing measured average (dots), and fitted curves.

Figure 4 shows the measured average electrical resistance increase of the samples stored at −10 °C for 40 weeks and the fitted curves on the measured data. Since the ranges of the measured absolute values are quite wide, a logarithmic scale was used to compare the results. 

The time of transient nucleation was determined when the average resistance of the samples increased by 10% (0.02 mΩ). This change was detectable since it was considerably over the repeatability error of the measurement (2%) and the deviation between samples (4%). According to this condition, the transient nucleation time was at 12 weeks for the reference samples and 22 weeks for the recrystallized samples, respectively. In the case of both samples, the average resistance changes showed an exponential increase. During the data fitting, the same exponential equation could be used but with different exponents:

(1) $$R_{ref}(-10{}^{\circ}C,t)=R_{0}\cdot \left(1-\frac{1-e^{0.036\cdot t}}{1000}\right)$$

(2) $$R_{rec}(-10{}^{\circ}C,t)=R_{0}\cdot \left(1-\frac{1-e^{0.023\cdot t}}{1000}\right)$$

where *R*_0_ is the initial resistance value of the samples (0.2 mΩ) and *t* is time (days). The exponent of the recrystallized samples (Equation (2)) is 64 % of the exponent of the reference samples (Equation (1)). At the end of the test, the average resistance of the reference samples was 2.6 mΩ (a 13-fold increase compared to the initial value, 0.2 mΩ). In contrast, in the case of the recrystallized samples, it was only 0.35 mΩ (a 1.75-fold increase compared to the initial value, 0.2 mΩ). Some of the reference samples started to lose their integrity (decompose) after 38 weeks. This usually occurred after a 20–30 times resistance increase compared to the initial resistance value (0.2 mΩ). The highest measured resistance was 11 mΩ (a 55-fold increase compared to the initial value, 0.2 mΩ) after 36 weeks of storage at −10 °C. The saturation phase of the transition was never reached during the test.

**Figure 5:**
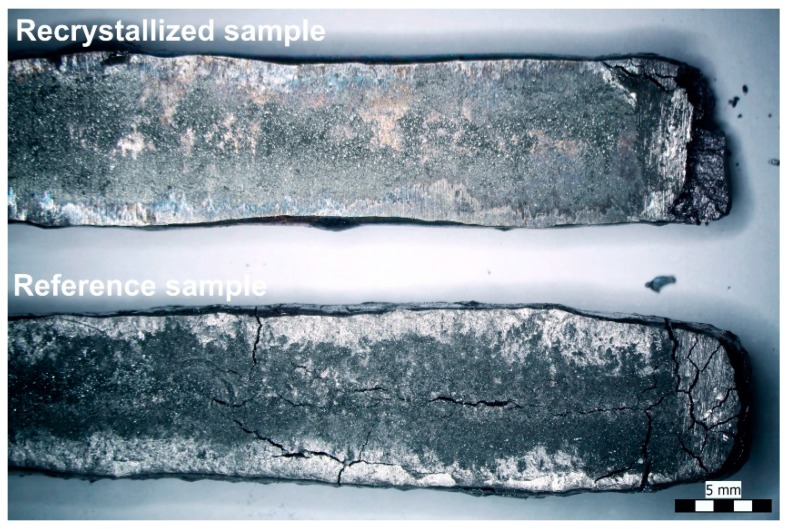
A reference and a recrystallized sample after being stored at −10 °C for 40 weeks.

The different degrees of allotropic transition in the case of different samples were also visually observable. Figure 5 shows a reference and a recrystallized sample after storage at −10 °C for 40 weeks. 

In the case of the recrystallized samples, marks of the transition (mainly cracking) were usually found only at the edges/ends, while in the case of the reference samples, marks of the transition were visible on the whole surface. This could be explained by the fact that annealing of the samples resulted in not only an increase in the grain size but also removed some of the strain energy from the surface, which was stored during the lamination.

**Figure 6:**
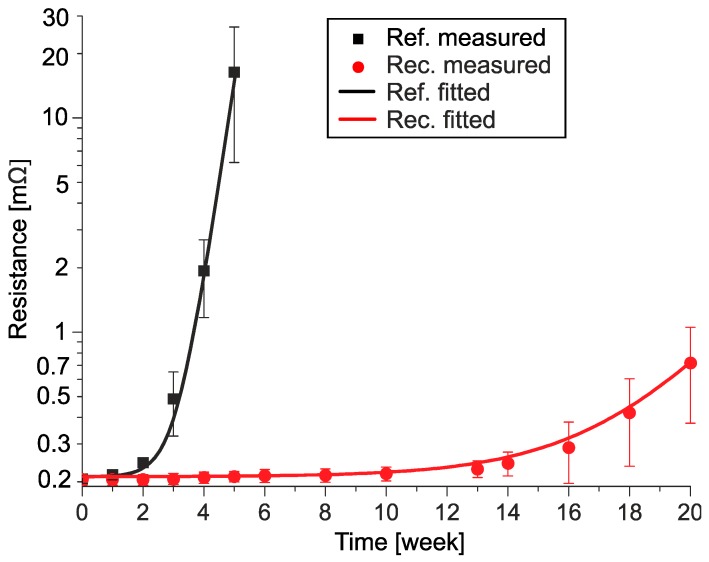
Plot of the electrical resistance changes in the reference (black) and recrystallized (red) samples at −20 °C, showing measured averages (dots), and fitted curves.

Figure 6 shows the measured average electrical resistance increase of the samples stored at −20 °C for 20 weeks and the fitted curves on the measured data. Here, the transient nucleation time was only 1 week for the reference samples and 10 weeks for the recrystallized samples, respectively. The lower temperature test resulted in even more noticeable differences between the sample types than the −10 °C test. In the case of both samples, the average resistance changes showed an exponential increase. During the data fitting, the same exponential equation could be used but with different exponents:

(3) $$R_{ref}(-20{}^{\circ}C,t)=R_{0}\cdot \left(1-\frac{1-e^{0.32\cdot t}}{1000}\right)$$

(4) $$R_{rec}(-20{}^{\circ}C,t)=R_{0}\cdot \left(1-\frac{1-e^{0.056\cdot t}}{1000}\right)$$

In this case, the difference between the exponents is even more considerable than at −10 °C. The exponent of the recrystallized samples (Equation (3)) is only 17% of the exponent of the reference samples (Equation (1)). The reference samples reached an average resistance of 16 mΩ (an 80-fold increase compared to the initial value, 0.2 mΩ) in 5 weeks, and all samples lost their integrity during the 6^th^ week of the test. The highest detected resistance was 80 mΩ (a 400-fold increase compared to the initial value, 0.2 mΩ). The average resistance of the recrystallized samples was 0.71 mΩ (a 3.5-fold increase compared to the initial value, 0.2 mΩ) after 20 weeks at −20 °C. The saturation phase of the transition was not detected during the test. By comparing the exponents of the two test temperatures, it can be concluded that the exponent at −20 °C is increased by 10 times in the case of the reference samples, but only by 2.5 times in the case of the recrystallized samples.

The process and spreading of the allotropic transition of β-Sn were investigated, to explain the considerable differences between the reference and recrystallized samples. For this purpose, the storage test at −10 °C was also performed on previously cross-sectioned solder bars. The cross-sectioned parts of the sample bars were embedded into epoxy resin and were sputter-coated with Au to prevent the corrosion of the exposed internal structure of the sample during the test. 

**Figure 7:**
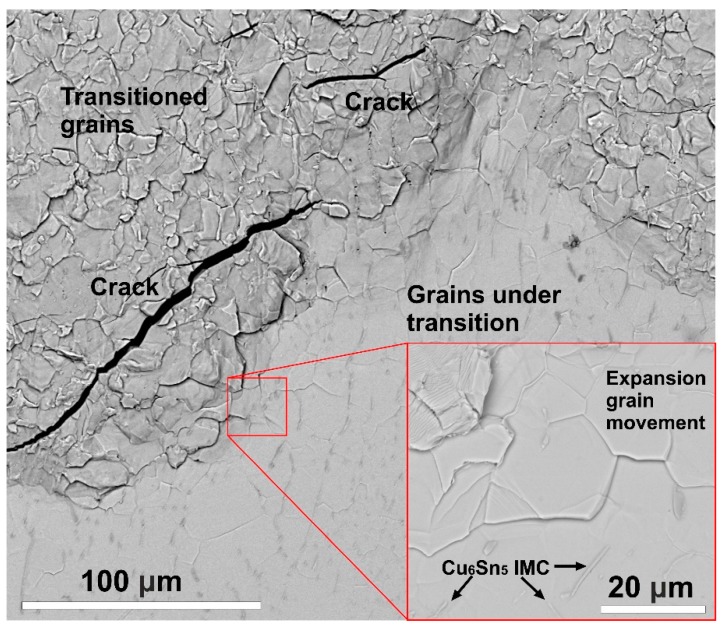
Scanning Electron Microscope (SEM) micrograph showing the different states of allotropic transition below the surface of a reference sample inoculated with InSb and stored at −10 °C for 100 days.

The process of transition is presented in Figure 7. The transition develops from the surface towards the bulk of the sample, and it can be divided into three stages. Initially, the grains under transition start to expand, which causes the grains to slide on each other. In the middle of the transition process, most of the grains are already transitioned, but separation has not started yet. At the end, the separation of the transitioned grains (results in decomposition of the whole sample body) begins with the formation of microcracks. In the case of the recrystallized samples, the separation of the grain occurred mainly at the edges and ends of the samples (Figure 5), but the whole sample body was not observed to decompose.

**Figure 8:**
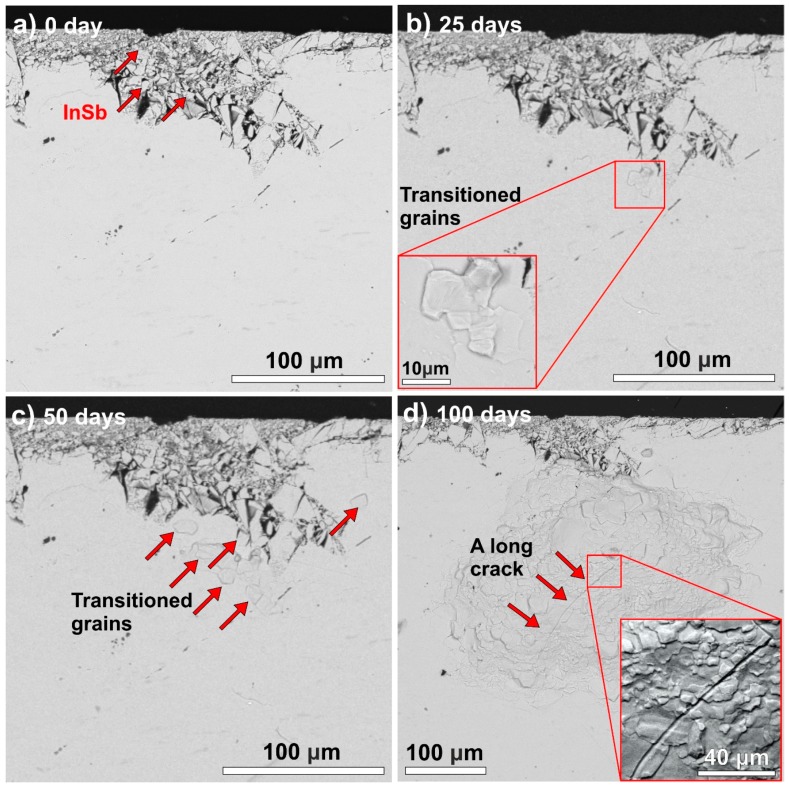
SEM micrographs showing the progress of the allotropic transition inside a reference body of samples inoculated with InSb at −10 °C: (**a**) 0 days; (**b**) 25 days; (**c**) 50 days; (**d**) 100 days.

The spread of the allotropic transition in the case of InSb inoculation is presented in Figure 8. The flake-like particles on the surface of the sample are InSb inoculator particles (Figure 8a). After 25 days of storage at −10 °C, the sliding of the grains is already visible close to the surface of the sample (Figure 8b). After 50 days of storage at −10 °C, more and more grains started to transform, and the transition spread vertically and horizontally as well (Figure 8c). The results after 100 days of storage at −10 °C show that in the case of InSb inoculation, the spread of the transition is as fast vertically as horizontally (Figure 8d). Most of the grains are already transitioned in the affected area, and a long crack is already visible as well. This result is interesting since, in the case of α-Sn inoculation (when α-Sn powder is used as an inoculator), the development of the allotropic transition is much faster horizontally then vertically [8]. In the case of InSb inoculation, the InSb might diffuse into the sample body, and this could speed up the transition vertically. However, more investigation is necessary to clarify this phenomenon. 

Previously, the growth kinetics of the β-Sn to α-Sn transition was determined mainly by the impurities and/or alloying elements (e.g., Ag and Cu) in the Sn; the size of the β-Sn particles; the tin grain size; the temperature; and the thermomechanical history before the transformation [10,12,15,22,29]. In our study, there was one order of magnitude grain size difference between the reference and the recrystallized samples (Figure 3). Taking into consideration that the samples used in the experiment were mechanically treated, which causes the grains to deform [30], the influence of recrystallization on the β-Sn to α-Sn transition might be explained as follows. Any deformation can introduce lattice defects like vacancies and dislocations. It increases the strain energy. The material itself tries to remove the increased energy and restore the original microstructure by the recovery. During it, annihilation and rearrangement of the dislocations occur [31]. The recovery usually takes place only in the bulk of the grains, rarely reaching the grain boundaries, and in normal circumstances, this process is prolonged.

However, annealing (at 180 °C/72 h) results in solid-state diffusion and causes the fast recrystallization of the material. Firstly, recrystallization decreases the number of vacancies and dislocations [31]. However, the material is still rich in grain boundaries, which are in a metastable state. Further annealing initiates the grain growth (smaller grain elimination) [32]. The grain growth is also affected by mechanical treatment (like applied axial lamination), which is attributed to strain energy created by mechanical treatment. Strain energy can influence abnormal grain growth: bimodal grain size distribution with small and large grains can develop. In our work, recrystallization of the samples was proven, since during the annealing, the average grain size grew 10 times (Figure 3). According to the process of recrystallization, it can be assumed that the number of vacancies and dislocations decreased too. Therefore, the suppressing effect of recrystallization on the allotropic transition of β-Sn to α-Sn can be explained by the decreased defects in the crystal-structure-like grain boundaries, vacancies, and dislocations, where heterogeneous nucleation could occur.

## 4. Conclusions

The effect of the recrystallization of 99.3Sn–0.7Cu wt. % solder alloy on the allotropic transition of β to α-Sn was investigated. InSb inoculation, and low temperature storages were used to enhance the allotropic transition. In the case of all sample types and test conditions, the resistance increase could be expressed with the same exponential equation but with different exponents. The transient nucleation time was 2 and 10 times shorter, and the exponent of the resistance increase was 2.5 and 10 times higher at −20 °C than at −10 °C test. It was found that recrystallization suppressed the tin pest phenomenon considerably. The exponents of the resistance changes in the case of recrystallized samples were only 64% (−10 °C) and 17% (−20 °C) of the exponents in the case of the reference samples. The positive effect of recrystallization can be explained by the grain boundary decrease (grain size increase) and by the likely decrease of vacancies and dislocations where heterogeneous nucleation could occur. Furthermore, it was found that in the case of InSb inoculation, the spread of the transition is as fast vertically as horizontally. The diffusion of InSb into the sample body might cause this, where the InSb could speed up the transition. This could explain the relatively fast decomposition of the samples. Further research is necessary to obtain more information on the effect of grain structure on the allotropic transition of β to α-Sn.
